# Notch Ligand DLL4 Alleviates Allergic Airway Inflammation *via* Induction of a Homeostatic Regulatory Pathway

**DOI:** 10.1038/srep43535

**Published:** 2017-03-06

**Authors:** Miao-Tzu Huang, Yi-Lien Chen, Chia-I Lien, Wei-Liang Liu, Li-Chung Hsu, Hideo Yagita, Bor-Luen Chiang

**Affiliations:** 1 Department of Medical Research, National Taiwan University Hospital, Taipei, 100, Taiwan; 2 Department of Pediatrics, National Taiwan University Hospital, Taipei, 100, Taiwan; 3 Graduate Institute of Clinical Medicine, School of Medicine, National Taiwan University, Taipei, 100, Taiwan; 4 Graduate Institute of Molecular Medicine, School of Medicine, National Taiwan University, Taipei, 100, Taiwan; 5 National Mosquito-Borne Diseases Control Research Center, National Health Research Institutes, Miaoli, 35053, Taiwan; 6 Department of Immunology, Juntendo University School of Medicine, Tokyo, Japan

## Abstract

Notch is a pleiotropic signaling family that has been implicated in pathogenesis of allergic airway diseases; however, the distinct function of individual Notch ligands remains elusive. We investigated whether Notch ligands, Jagged1 and DLL4, exert differential effects in OVA-induced allergic asthma. We found that whilst Jagged1 inhibition mitigated Th2-dominated airway inflammation, blockage of DLL4 aggravated the Th2-mediated asthma phenotypes. Additionally, Jagged1 signaling blockage enhanced IL-17 production and neutrophilic airway infiltration. *In vitro*, exogenous Jagged1 induced Th2-skewed responses, whereas augmented DLL4 signaling displayed a dual role by promoting expansion of both Tregs and Th17. *In vivo*, DLL4 blockage impaired Treg differentiation which plausibly resulted in exaggerated asthma phenotypes. On the contrary, administration of DLL4-expressing antigen-presenting cells promoted endogenous Treg expansion and ameliorated the allergic responses. Therefore, whilst Jagged1 induces Th2-skewed inflammation, DLL4 elicits an essential self-regulatory mechanism *via* Treg-mediated pathway that counterbalances Jagged1-induced Th2 responses and facilitates resolution of the airway inflammation to restore homeostasis. These findings uncover a disparate function of Jagged1 and DLL4 in allergic airway diseases, hinting feasibility of Notch ligand-specific targeting in therapy of allergic airway diseases.

Allergic asthma is characterized by recurrent inflammatory episodes of the airways towards harmless environmental factors. The resulting pathological changes of mucus metaplasia, increased subepithelial matrix deposition, and airway smooth muscle hypertrophy/hyperplasia cumulatively lead to airway hyperresponsiveness, irreversible airway remodelling and obstruction^1^,^2^. In addition to being a genetically predisposed disease^3^, allergic asthma has long been regarded as a Th2-skewed immune reaction causally attributed to insufficient Treg immune regulation^4^,^5^ and re-introduction of Tregs into asthmatic animals was able to suppress the allergic responses and revert the asthma phenotypes^6^,^7^,^8^.

The mammalian Notch family includes four Notch receptors, Notch-1, -2, -3 and -4 and five Notch ligands, Jagged-1, -2, and Delta-like (DLL)-1, -3 and -4. Upon ligand binding of the Notch, proteolytic cleavage of the transmembrane domain by γ-secretases releases the intracellular domain (NICD), which subsequently translocates into the nucleus to bind to the PBPJκ/CSL and together with the proteins of the Mastermind-like family, forms the transcriptional activation complex for the downstream target genes such as the HES family^9^. Among its pleiotropic functions in development and tissue renewal, Notch has been shown to regulate differentiation of literally almost all T-helper (Th) subsets, including Th1, Th2, Th9, Th17 and regulatory T cells (Tregs) via the induction of lineage-specific transcription factors as well as the signature products of distinct T-cell lineages^10^,^11^. However, with the common downstream canonical signaling events irrespective of individual Notch receptor-ligand pairs, how does Notch do it all? There are studies proposing a context-dependent mechanism wherein Notch acts as a signaling hub to integrate additional signals originated from the adjacent milieu, such as those derived from membrane receptors and cytokines. Alternatively, studies have also showed that engagement of distinct Notch ligands elicits signals differentially skewing Th subset differentiation. In this theory, ligand-specific signaling outcome is conceivably orchestrated by characteristic expression pattern of individual Notch family members under distinct inflammatory circumstances. The latter proposition undoubtedly lays the basis for therapeutic approaches exploiting ligand-specific targeting of Notch signaling pathway. In support of this theory, gain-of-function studies have showed that while DLL engagement induces Th1 differentiation, Jagged generates signals that favor the alternate Th2 fates^12^,^13^,^14^.

γ-Secretase inhibitors (GSI) are non-specific inhibitors of Notch signaling pathway. Clinical trials and experimental studies in various cancers and immune-mediated diseases have shown a therapeutic potential of GSI. In experimental allergic airway diseases, administration of GSI caused a general suppression of the asthma phenotypes^15^. In addition, GSI-treated CD4^+^ or CD8^+^ T cells failed to restore airway inflammation in IL-4- or CD8-deficient mice, or to enhance the responses in WT mice^16^,^17^. Nevertheless, non-specific suppression of Notch may cause unfavorable outcomes considering the pleiotropic functions that have been attributed to Notch in various biological processes. The potential drawbacks in this concern together with studies that showed a more effective modification of Notch signaling by using receptor/ligand-specific approaches support therapeutic advantages of specific Notch targeting^18^.

In this study, we investigated the distinct role of Jagged1 *vs* DLL4 in allergic airway diseases by using the OVA-induced murine asthma model. We found that whilst Jagged1 contributed to allergic Th2 responses, DLL4 counteracted the Th2 responses *via* the induction of endogenous Treg-mediated regulatory pathway which conceivably constitutes an inducible and essential homeostatic mechanism during allergic airway inflammation.

## Results

### Upregulated Notch expression during allergic airway inflammation

**Figure 1:**
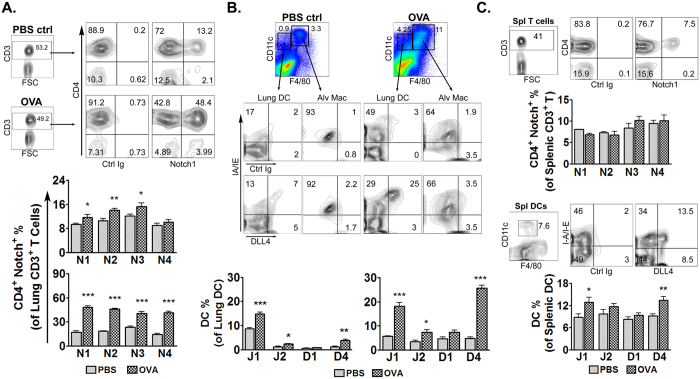
Allergen sensitization and challenge enhanced expression of Notch receptors and ligands in infiltrated pulmonary leukocytes. 6 w/o female BALB/c mice were sensitized and challenged by OVA as described in the Methods. Pulmonary leukocytes and splenocytes were isolated and analyzed by FACS for expression of Notch receptors and ligands. (**A**) Notch-expressing CD4^+^ lung T cells before (*upper*) and after (*bottom*) OVA challenge; Representative FACS plots showed Notch expression of lung CD4^+^ T cells isolated from PBS control and asthmatic mice. (**B**) (*Upper*) FACS plots showed expression of Notch ligands by lung DCs. Lung DCs were gated by expression of CD11c, after excluding F4/80-expressing macrophages; (*Bottom*) Notch ligand-expressing profile of lung DCs before (*left*) and after (*right*) OVA challenge. (**C**) Expression profile of Notchs by splenic CD4^+^ T cells and DCs. FACS plots showed representative Notch and Notch ligand expression by splenic T cells and DCs, respectively. Data were representative of 3 independent experiments; N = 4–6 mice/group. Comparisons were made by un-paired t-test. *P < 0.05; **P < 0.01; ***P < 0.001.

To investigate the distinct role of Notch in pathogenesis of allergic airway diseases, expression profile of Notch in pulmonary leukocytes was examined. We found that OVA-sensitization alone induced expression of Notch1, Notch2 and Notch3 in pulmonary CD4^+^ T cells compared to PBS treatment. The enhanced expression pattern of the Notch receptors in CD4^+^ T cells of the OVA-sensitized mice was further upregulated after aerosol OVA challenge (Fig. 1A). In parallel, OVA sensitization induced the expression of Jagged1, DLL4 and, to a lesser extent, Jagged2 in pulmonary CD11c^+^DCs. After aerosol OVA challenge, the DC populations expressing Jagged1, Jagged2 and, much more prominently, DLL4 were further increased (Fig. 1B). On the contrary, CD11c^+^ alveolar macrophages expressed only low levels of Notch ligands despite OVA challenge, indicating DCs as the main expressers of Notch ligands and the key player in pathogenesis of allergic airway diseases.

The expression profile of the Notch family components in splenic CD4^+^ T cells and CD11c^+^DCs was also examined (Fig. 1C). In contrast to their lung-infiltrating counterparts, splenic CD4^+^ T cells showed comparable expression of the Notch receptors despite OVA sensitization and challenge compared to PBS controls, whereas OVA treatment moderately increased the Jagged1- and DLL4-expressing DC populations of the spleens.

### DLL4 and Jagged1 signaling blockage leads to different asthma phenotypes

**Figure 2:**
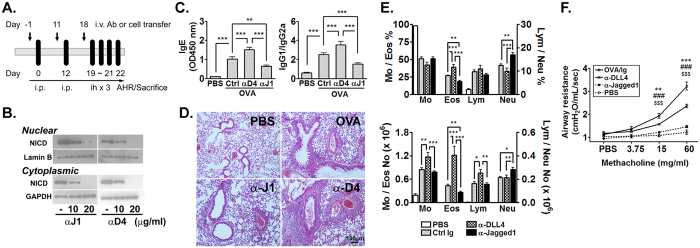
Differential regulation of asthma phenotypes by DLL4 and Jagged1. BALB/c mice were sensitized and challenged with OVA as described. In indicated groups, DLL4 or Jagged1 blocking Ab were given 1 hr before sensitization and challenges. Mice receiving PBS sensitization and challenge and mice given isotype control Ab before OVA sensitization and challenge were used as negative and positive controls, respectively. Serum was collected for Ab detection at one wk after the 2^nd^ sensitization. Bronchoalveolar lavage and histologic studies of the lungs were performed after aerosol OVA challenge. (**A**) Schematic diagram showing the experimental design of the OVA-induced asthma model. (**B**) NICD expression in nuclear and cytoplasmic extract after stimulation of the splenocytes in the presence of Jagged1 and DLL4 blocking Ab at 10 and 20 μg/mL. (**C**) Serum OVA-specific Ab detected by ELISA; (**D**) Microphotographs showing H&E staining of the lungs; (**E**) Differential counts of BALF leukocytes enumerated by staining characteristics of individual leukocyte populations. A total of 500 cells per cytospin were counted. N = 6–8 mice/group. Data were representative of 4 independent experiments. Comparisons between groups were made by one-way ANOVA. *P < 0.05; **P < 0.01; ***P < 0.001; (**F**) Airway resistance was recorded through an intratracheal cannula while the animals were exposed to increasing concentrations of methacholine (0~60 mg/mL) under anesthesia. N = 6–8 mice/group. Data were representative of 4 independent experiments. Analysis was made by one-way ANOVA. * and ^#^Comparison of OVA group to α-DLL4 and α-Jagged1 groups, respectively; ^$^α-DLL4 compared to α-Jagged1 group. **P < 0.01; ***, ^###^ and ^$$$^*P* < 0.001.

To investigate the differential effects of Jagged1 and DLL4 in allergic airway inflammation, selective blockage of Jagged1 and DLL4 was conducted by treating the mice with Jagged1- and DLL4-specific blocking Abs along the course of OVA sensitization/challenge (Fig. 2A). The blocking effect of the Abs was confirmed by decreased NICD expression in both the nuclear and cytoplasmic lysates of stimulated splenic T cells (Fig. 2B). The protocol with Abs administered both before OVA sensitization and challenge was adopted since we observed only a lesser or marginal effect when Abs were given solely before sensitization or challenge. In our OVA-induced murine asthma model, OVA sensitization induced the production of OVA-specific IgE and a Th2-skewed IgG subclass profile wherein the levels of IgG1 prevailed that of IgG2a (Fig. 2C). In mice treated with DLL4 blocking Ab, OVA-specific IgE and the Th2-skewed IgG profile were even more enhanced compared to control Ig-treated OVA mice. In contrast, Jagged1 blockage significantly mitigated the characteristic IgE and IgG Ab profile.

Histologic studies of the lungs showed significantly increased cell infiltration in DLL4 Ab-treated mice compared to Ig- and Jagged1 Ab-treated mice (Fig. 2D). However, whilst blockage of Jagged1 signaling alleviated the induction of OVA-specific Ab profile, lung histology of these mice displayed comparable leukocyte infiltration as the Ig-treated controls, implying disparate effects of Jagged1-derived signals in allergen-induced Ab production and leukocyte recruitment. In accordance with the histologic findings, DLL4 Ab-treated mice exhibited significantly increased BALF infiltrates of monocyte, eosinophils and lymphocytes compared to their Ig- and Jagged1 Ab-treated counterparts (Fig. 2E). On the other hand, although blockage of Jagged1 hampered eosinophil recruitment into the lungs, neutrophil infiltration in these mice was significantly increased. Therefore, the comparable leukocyte infiltration between Jagged1Ab-treated mice and Ig-treated controls, as observed in histologic studies, was composed of different leukocyte populations. Decreased BALF eosinophils after Jagged1 blockage was somehow compensated by increased neutrophil trafficking, whilst the lack of DLL4 signals elicited the opposite effects by enhancing eosinophil recruitment but not neutrophil trafficking.

Airway hyperresponsiveness of these mice were accessed by challenging the mice with escalating concentrations of aerosol methacholine (Fig. 2F). As can be expected, Ig-treated OVA mice displayed prompt and increased airway responsiveness to methacholine compared to PBS controls. In mice treated with DLL4 Ab, airway hyperresponsiveness was significantly aggravated. In contrast, inhibition of Jagged1 improved airway hyperresponsiveness to levels of the PBS controls. It is noteworthy that severe bronchospasm developed in ~30–40% of the mice treated with DLL4 Ab, which led to mortality of these mice during the process of methacholine challenge. The aggravated asthma phenotypes after DLL4 blockage suggest probable beneficial effects of DLL4 during allergic airway inflammation.

### DLL4 and Jagged1 orchestrate divergent cytokine secretion in allergic airway diseases

**Figure 3:**
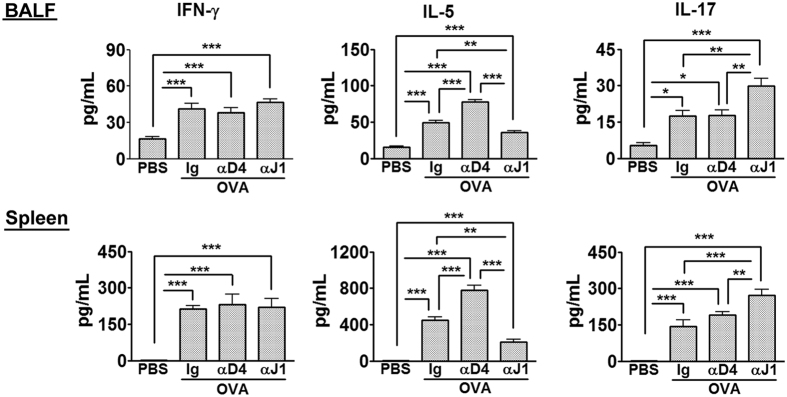
Blockage of DLL4 signaling enhanced Th2-inflammation whereas Jagged1 signaling blockage released IL-17 response. BALF and splenocytes were collected at one day after aerosol OVA challenge. Splenocytes were stimulated with 200 μg/ml OVA for 48 hrs. Cytokines in (**A**) BALF and (**B**) culture supernatants of splenocytes were analyzed by ELISA. N = 4–6 mice/group. Data were representative of 3 independent experiments. Comparisons between groups were made by one-way ANOVA. ***P < 0.001.

BALF collected after OVA challenge was examined for cytokine expression (Fig. 3A). Increased IFN-γ, IL-5 and, at lower levels, IL-17 were found in BALF of essentially all OVA-treated mice compared to PBS controls. In Notch-ligand blockage groups, whilst DLL4 blockage selectively enhanced IL-5 production, inhibition of Jagged1 resulted in decreased IL-5 and increased IL-17 secretion compared to the other two treatments. These findings indicate divergent functions of DLL4 and Jagged1 in the progression of allergic airway inflammation. In addition, cytokine secretion was also examined after OVA re-stimulation of the splenocytes (Fig. 3B). Similar to what was found in BALF, splenocytes isolated from DLL4 Ab-treated mice selectively produced more IL-5, whereas splenocytes of the Jagged1 blockage group exhibited decreased IL-5 and enhanced IL-17 secretion.

Based on the above findings and previous literatures, we speculated a counterbalanced effect of Jagged1 and DLL4 in pathogenesis of allergic airway diseases. Whilst Jagged1-derived signals are known to preferentially induce Th2 responses, DLL4 has been shown to induce Th17 responses. Therefore, blockage of either Jagged1 or DLL4 would likely unleash the effects of the opposite pathway. To this aspect in our current study, inhibition of DLL4 will provide permissive conditions for Jagged1 signaling that cumulated in aggravated and, in one third of the mice, lethal allergic airway inflammation. However, based on our current results thus far, the “counterbalancing” theory could not fully explain the seemingly paradoxical results on neutrophilic airway inflammation, as can be induced by DLL4-elicited IL-17, and the otherwise mitigated asthma phenotypes after Jagged1signaling blockage. It is possible that other operational mechanisms derived from DLL4 after inhibition of Jagged1 are as yet unidentified and it is our interests to explore in this study.

### DLL4 and Jagged1 exert differential regulation of Th subset differentiation

**Figure 4:**
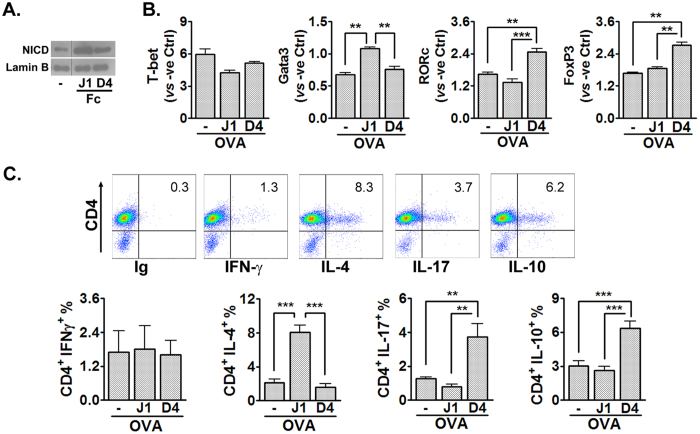
DLL4 and Jagged1 exerted distinct regulation on Th subset development. CD4^+^ T cells were isolated from 6 w/o DO11.10 mice and stimulated with OVA_323–339_ peptide-pulsed irradiated BALB/c APCs in the presence of Fc-conjugated Jagged1 and DLL4 recombinant proteins. (**A**) Nuclear NICD expression evaluated by Western blotting at 16 hr after stimulation. At 48 hr after stimulation, cells were harvested and analyzed for Th subset development. (**B**) Quantification of main Th subset transcription factors by qPCR; (**C**) cytokine production was detected by intracellular FACS staining. (*Upper*) representative FACS plots showing intracellular cytokine expression of the CD4^+^ T cells; (*Bottom*) percentage of individual cytokine-expressing CD4^+^ T-cell subsets. Data represent one of three independent experiments. Comparisons between groups were made by one-way ANOVA. **P < 0.01; ***P < 0.001.

To delineate further the effector mechanisms of DLL4 and Jagged1 signaling, we performed a series of *in vitro* studies wherein CD4^+^ T cells were stimulated in the presence of Jagged1 and DLL4 recombinant proteins. The additive effect of the Jagged1 and DLL4 proteins was verified by increased NICD expression of the stimulated splenocytes (Fig. 4A). Gene expression of key Th subset transcription factors was analyzed after 48-hr stimulation (Fig. 4B). The data showed comparable T-bet expression irrespective of the status of Jagged1 or DLL4 signaling; nonetheless, Gata3 was induced by augmented Jagged1 but not DLL4 signaling. In addition, whilst RORc expression was significantly increased by addition of DLL4, augmented DLL4 signaling also enhanced FoxP3 expression, suggesting a dual role of DLL4 in Th subset differentiation. The particular Th subtypes as regulated by exogenous Jagged1 and DLL4 were also analyzed by intracellular cytokine staining (Fig. 4C). In accordance with the results of Th subset transcription factors, Jagged1-derived signals selectively induced IL-4-secreting Th-cells. On the other hand, DLL4 promoted both IL-17- as well as IL10-secreting Th-cells. Collectively, the *in vitro* data demonstrated a Th2-promoting function of Jagged1 and a dual effect of DLL4-derived signals in Th17 and Treg-cell differentiation.

These findings not only support our speculation that Jagged1 and DLL4 generated mutually counterbalanced signals, they also prompted that the aggravated asthma severity after DLL4 blockage can be attributed to the DLL4-elicited regulatory pathway that prevails the Th17-mediated inflammation in the asthma model.

### DLL4 induces expansion of endogenous Tregs and ameliorates allergic airway inflammation

**Figure 5:**
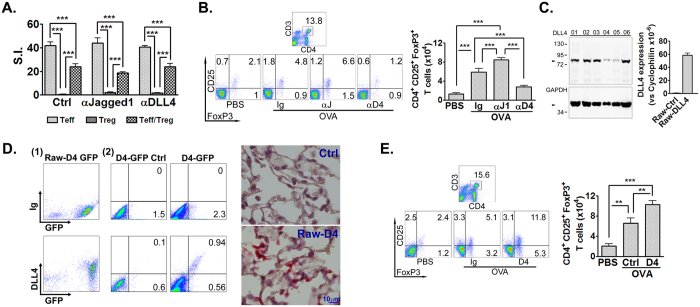
DLL4 induced endogenous Tregs during allergic airway inflammation. CD4^+^CD25^+^ Tregs and CD4^+^CD25^-^ effector T cells (Teff) were isolated from the spleens of DO11.10 mice and stimulated with 200 μg/mL OVA in the presence of α-DLL4 or α-Jagged1 Ab (20 μg/mL). (**A**) Suppression of Teff proliferation by CD4^+^CD25^+^ Tregs in response to OVA stimulation. Data are representative of three separate experiments. Comparison was made by one-way ANOVA. ***P < 0.001. To investigate the induction of pulmonary Tregs, mice were sensitized and challenged with OVA as described in the Methods. When indicated, mice were given control Ig, α-DLL4 or α-Jagged1 Ab *via i.v.* before OVA sensitization and challenges. (**B**) CD4^+^CD25^+^ FoxP3^+^ T cells of the lungs after OVA challenge were detected by FACS. Data represented one of three independent experiments. N = 5 mice/group. Comparisons between groups were made by one-way ANOVA. ***P < 0.001. In parallel, mice were given DLL4-overexpressing Raw264.7 (Raw-DLL4, D4) or Raw264.7 transfected with empty vector (Raw-Ctrl, Ctrl) before OVA sensitization and challenges. (**C**) (*left*) DLL4 expression by Raw-DLL4 clones were examined by Western blotting; (*right*) DLL4 expression by the Raw-DLL4 and Raw-Ctrl clones selected for *in vivo* transfer was quantified by qPCR. To verify the entry of Raw-DLL4 into the lungs, Raw-DLL4 was further overexpressed with GFP as described in the Methods. Pulmonary leukocytes and lung tissues were collected at 30 min and 2hr after transfer. (**D**) (*left*) FACS plots showed GFP-overexpressing Raw-DLL4 before transfer and in the lungs at 2 hr after transfer; (*right*) microphotographs at 2 hr showed the presence of Raw-DLL4 in the lungs as labeled in red by GFP immunostaining. (**E**) Pulmonary CD4^+^CD25^+^ FoxP3^+^ Tregs after Raw-DLL4 transfer were detected by FACS. Data represented one of two independent experiments. N = 6 mice/group. Comparisons between groups were made by one-way ANOVA. **P < 0.01; ***P < 0.001.

The necessity of DLL4 in immune suppressive function of Tregs was investigated by antigen-stimulated proliferation assay which showed that blockage of DLL4 did not affect the immune suppressive function of Tregs, and neither did Jagged1 blockage (Fig. 5A). The role of DLL4 in endogenous Treg induction was next envisaged in the murine asthma model (Fig. 5B). We found that pulmonary Tregs was significantly increased in asthmatic mice compared to PBS controls. In the setting of DLL4 blockage, Treg population was significantly diminished; however, the lack of Jagged1 signals resulted in enhanced Treg expansion. Therefore, exacerbation of the allergic responses after blockage of DLL4 was not due to impaired Treg immune suppression, but was attributed to insufficient Treg differentiation.

Whilst kindred of inflammatory leukocytes cooperated to propagate the inflammatory response, induction of endogenous Tregs and/or recruitment of Tregs into the lungs worked simultaneously alongside the progression of allergic inflammation. We speculated that the emergence of endogenous Treg population in the lungs during allergic airway inflammation constituted an inducible homeostatic mechanism to resolve the allergic responses and inadequate induction of this regulatory population would hence lead to aggravated pathology.

**Figure 6:**
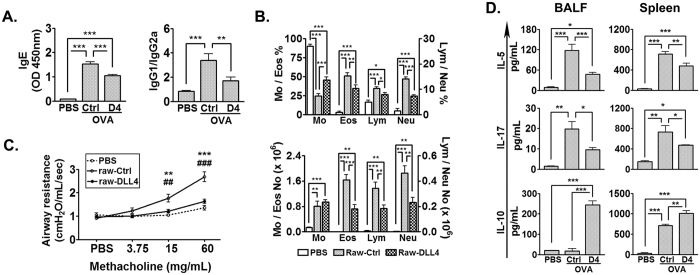
DLL4 ameliorated allergic airway inflammation. Mice were OVA-sensitized and challenged as described in the Methods. In indicated groups, mice were given Raw-DLL4 or Raw-Ctrl *via* i.v. before OVA sensitization and challenge. (**A**) Serum OVA-specific Ab detected by ELISA; (**B**) Differential BALF leukocyte counts. A total of 500 cells per cytospin were counted. N = 6 mice/group. Data were representative of two independent experiments. Comparisons between groups were made by one-way ANOVA. *P < 0.05; **P < 0.01; ***P < 0.001; (**C**) Airway resistance was measured by exposing the mice to escalating concentrations of methacholine (0~60 mg/mL). N = 6–8 mice/group. Data were representative of two independent experiments and analyzed by one-way ANOVA. ^#^PBS controls compared to Raw-Ctrl; *Raw-DLL4 group compared to Raw-Ctrl. ^##^ and **P < 0.01; ^###^ and ***P < 0.001; (**D**) Cytokine expression in BALF and splenocyte culture supernatants was analyzed by ELISA. N = 6 mice/group. Data were representative of two independent experiments. Comparisons between groups were made by one-way ANOVA. *P < 0.05; **P < 0.01; ***P < 0.001.

To verify the *in vivo* regulatory effect of DLL4, mice were given DLL4-overexpressing Raw-DLL4 before OVA sensitization and challenge (Fig. 5C). Raw-DLL4 cells can be detected in the lung as early as 30 min after transfer and trafficking of the Raw-DLL4 into the lungs continued when observed at 2 hr (Fig. 5D). BALF Tregs analyzed after OVA challenge showed a significant induction of Tregs after Raw-DLL4 transfer (Fig. 5E). The OVA-specific IgE and Th2-IgG profile was improved in mice receiving Raw-DLL4 transfer compared to OVA mice receiving Raw-Ctrl (Fig. 6A). Additionally, eosinophilic airway infiltration and airway hyperresponsiveness were both ameliorated after Raw-DLL4 transfer (Fig. 6B,C). Noticeably, BALF neutrophils were also decreased after Raw-DLL4 transfer. Cytokine profile in BALF and spleen demonstrated concordant results; whilst expression of IL-5 and IL-17 were decreased in Raw-DLL4-transferred mice, IL-10 production was increased (Fig. 6D). The potential detrimental effects of IL-17 production and neutrophilic airway trafficking, as can be induced by DLL4, were not evoked by Raw-DLL4 transfer, supporting a dominant role of DLL4-mediated Treg induction *in vivo*. Collectively, these data support a Treg-dependent immune regulatory role of DLL4 in allergic airway diseases.

## Discussion

Our study demonstrated the divergent functions of Jagged1 and DLL4 in pathogenesis of allergic airway inflammation. Whilst Jagged1 signaling blockage mitigated the Th2-dominated allergic responses, inhibition of DLL4 exaggerated the allergic phenotypes. We found that DLL4-derived signals ameliorated the allergic responses *via* induction of Treg-mediated regulatory pathway. It is worth noting that DLL4 also promoted Th17 differentiation; however, in the murine asthma model, Treg-mediated immune regulation, as induced by DLL4, overcame Th17-mediated inflammation, which conceivably led to aggravated allergic phenotypes after DLL4 blockage. It is plausible that allergic inflammation simultaneously generates homeostatic self-regulatory cues upon which DLL4 responses with signals favoring Treg differentiation over Th17. This speculation was supported by the findings that DLL4-overexpressing APCs enhanced Treg expansion and ameliorated the allergic pathology.

Although Notch is implicated in the differentiation program of all known Th subsets, there has been no consensus regarding the exact operation mechanisms that can be ascribed to explain how the various Notch family members are selectively called into effects in these diverse and often mutually exclusive processes. The signaling competence of unique Notch receptor-ligand pairs has been shown to be regulated by Fringe-mediated glycosylation in the Golgi^19^,^20^. Therefore, Notch receptors can be modulated to deliver signals selectively from particular ligands but not others before ligand-binding on the cell surface. Notch can also be activated following TCR stimulation without ligand engagement, albeit through yet unknown mechanisms^21^. These activation characteristics provide an operation mechanism for Notch to exert their effects in response to environmental cues during inflammation. Previous studies have reported a role for Jagged in promoting Th2-cell differentiation and inflammation^16^,^22^. However, in our current study, Jagged1 inhibition did not completely abrogate the allergic phenotypes but mitigated the intensity of the allergic responses. It is plausible that other molecules and signaling family operated in companion of Notch-Jagged1 in pathogenesis of allergic airway diseases. Therefore, Notch-Jagged1 signaling pair reacts in response to or in coordination with the environmental cues to modulate the overall inflammatory response. Nonetheless, it is also conceivable that Jagged2 molecule may also play a role despite the expression levels of Jagged2 in pulmonary leukocytes were extremely low in our current study.

On the other hand, DLL has been shown to generate signals that antagonize Jagged-induced Th2 inflammation^23^. In line with these findings, DLL4 was demonstrated to tune-down allergic airway inflammation by inducing Th2-cell apoptosis^24^ and administration of DLL1-Fc protein was shown to prevent allergic airway responsiveness by skewing the Th2 response to a Th1 phenotype^17^. In addition, the protective Th1-cell response to respiratory syncytial virus infection was switched to Th2-mediated allergic responses after inhibition of DLL4 signals^25^. Adding to the counterbalancing effects of the Jagged and DLL, our studies demonstrated an aggravated allergic phenotype after DLL4 inhibition that was attributed to insufficient Treg differentiation in the absence of DLL4 signals. Therefore, although Notch exerts function in a context-dependent manner, there is also the existence of ligand-specific functions that can be exploited for therapies of allergic airway diseases. It is conceivable that differential utilization of Notch ligands operates as a mechanism orchestrating the nature and extent of the immune activation *vs* regulation.

Notch has been shown to induce IL-10 production in established Th1 cells as an essential mechanism for self-regulation of Th1 immunity. In these studies, whilst IL-10 can be elicited by all four Notch receptors, the response was exclusively triggered by DLL4 but not Jagged^26^. On the role of DLL4 in Treg differentiation, our data have been contradictory to previous studies of different disease models. In experimental allergic encephalomyelitis, type 1 diabetes and graft-versus-host disease models, blockage of DLL4 was demonstrated to ameliorate disease severity through induction of Tregs^27^,^28^,^29^. In addition, whilst Tregs preferentially expressed Notch-3, -4 and Jagged1, DLL1 and DLL4^30^,^31^,^32^, Treg differentiation was induced following Notch3 activation and Jagged engagement^33^,^34^,^35^. The discrepancy can partly be reconciled by the context-dependent operation characteristic that orchestrates the overall outcome of Notch signaling; *i.e.* Th1/Th17-mediated pathologies induced by DLL4 in these disease models *vs* Th2-skewed responses attributed to Jagged1 in our asthma model.

The application of GSI to target Notch for deranged cell growth in various cancers has met some success in several clinical trials. It is expectable that manipulation of Notch signaling in immunologic compartments can be exploited to treat allergic airway diseases. Considering the functions of Notch in a plethora of biological processes and hence the potential side-effects derived from non-selective targeting, ligand-specific approaches pose an attractive and safer therapeutic strategy. To this aspect, inducing endogenous Tregs through DLL4 signaling will be applicable to prevention and resolution of allergic airway inflammation.

## Methods

### Animals

DO11.10 mice carrying an OVA_323–339_-specific TCR transgene and BALB/c mice were both purchased from the Jackson Laboratory (Bar Harbor, Maine, USA) and bred in the National Taiwan University animal facility under SPF conditions. Animal procedures were reviewed and approved by the National Taiwan University Animal Ethics Committee. All methods were performed in accordance with the relevant guidelines and regulations.

### OVA-induced allergic airway inflammation

6 w/o female BALB/c mice were sensitized by two i.p. injections of 50 and 20 μg OVA (Grade V; Sigma-Aldrich, St. Louis, MO) emulsified in 1% alum (Pierce Biotech, Rockford, IL, USA) on day (D) 0 and D12, followed by 30-min aerosol OVA (5%) challenge for three consecutive days since D19. In indicated groups, mice were given anti-DLL4 or anti-Jagged1 blocking Ab (10 mg/kg; BioXCell, NH, USA) or DLL4-expressing APCs (Raw-DLL4, 1.5 × 10^6^ cells/mouse) via tail vein 1 h before sensitization and challenge. OVA-sensitized mice receiving a hamster IgG isotype control Ab (Ig) or control APCs (Raw-Ctrl) were used as positive controls, whereas mice receiving PBS treatment were negative controls.

### Measurements of antibodies and cytokines

Serum OVA-specific Ab and cytokines in BALF and culture supernatants were measured by ELISA following manufacturer’s instruction (R&D systems and BioLegend) as previously described^38^.

### Bronchoalveolar lavage fluid (BALF) and differential leukocyte counts

Leukocytes in the BALF were pelleted and resuspended for cytospins (Shandon Cytospin 2; Thermo Scientific, MA, USA), followed by staining with Giemsa and May-Grünwald (Sigma-Aldrich). Differential leukocyte counts with a total cell count of 500 per cytospin were enumerated in a blinded fashion.

### Measurement of airway hyperresponsiveness (AHR)

Mice were anaesthetized and exposed to escalating concentrations of methacholine (0~60 mg/mL, Sigma-Aldrich) via a tracheal cannula and airway resistance was recorded by using the Buxco Pulmonary Mechanics System (Buxco Electronics, NC, USA). Lung resistance (cmH_2_O*Sec/mL) at individual methacholine concentration was recorded and compared with that of PBS after substracting baseline resistance.

### Isolation and characterization of pulmonary leukocytes

After PBS perfusion to expel intravascular blood content, the lungs were excised and minced, followed by digestion with 1 mg/mL collagenase IV and 0.5 mg/mL DNase I (Sigma-Aldrich) at 37 °C for 30 min. After digestion, the released leukocytes were resuspended in HBSS and centrifuged over 60% Percoll gradient (GE Healthcare, IL, USA) at 3200 rpm for 20 min. Leukocytes were collected from the Percoll/HBSS interface. Notch expression profile and Treg population were analyzed by FACS.

### Establishment of DLL4-overexpressing APC lines

Raw264.7 cells were grown to 80% confluence in 6-well plates before transfected with 2 μg pcDNA3.1-DLL4-Flag (Raw-DLL4), or pcDNA3.1-Flag empty vector (Raw-Ctrl) (GenScript, NJ, USA) by using Turbofect transfection reagents (Thermo-Scientific). At day 2 after transfection, culture media was replaced by G418-containing 10% DMEM (0.5 g/L, Invitrogen). G418-resistant clones were selected by limiting dilution in 96-well plates and expanded when reached confluent. For *in vivo* tracing of the transferred APCs, DLL4-overexpressing Raw cells were further transduced with pAS2-EGFP or pLKO-AS2 control pseudoviral particles (*RNAi*Core, Academia Sinica, TW), followed by selection with puromycin (2 μg/ml, Invitrogen). GFP-expressing Raw-DLL4 cells were examined by FACS or visualized by tissue immunostaining against GFP (Abcam, Cambridge, UK).

### Evaluation of Notch ligand-specific immune function

Splenic CD4^+^ T cells were isolated from DO11.10 mice by using magnetic bead-conjugated Ab per manufacturer’s negative selection protocol (Miltenyi, Bergisch Gladbach, Germany). 2 × 10^6^ CD4^+^ T cells were co-cultured with irradiated syngeneic APCs in the presence of OVA_323–339_ peptide (2.5 μg/mL). When indicated, Jagged1-Fc and DLL4-Fc recombinant proteins (10 μg/mL, R&D, Minneapolis, USA) were pre-coated onto 48-well plates at 4 °C for overnight before coculture. At 48 hr in culture, RNA lysate was collected for gene expression. Alternatively, cells were restimulated at 48 hr with PMA (100 ng/mL, Sigma-Aldrich), ionomycine (1 μg/mL) plus monesin for 6 hrs. Intracellular cytokine expression was detected by FACS.

### Evaluation for the efficacy of DLL4- and Jagged1-specific reagents

To validate the function of DLL4- and Jagged1-specific blocking Ab and recombinant proteins in modulating Notch signaling, splenocytes isolated from OVA-sensitized BALB/c mice were stimulated with 200 μg/mL OVA. When indicated, the stimulation was performed in the presence of anti-DLL4 or anti-Jagged1 Ab at 10 and 20 μg/mL (BioXCell); hamster IgG isotype Ab was used as the control. In parallel, Jagged1-Fc and DLL4-Fc recombinant proteins (10 μg/mL, R&D, Minneapolis, USA) were pre-coated onto 48-well tissue culture plates at 4 °C for overnight before the stimulation. At 16 hr in culture, protein lysates were collected for NICD Western blotting.

### Proliferation assay

CD4^+^CD25^+^ Tregs and CD4^+^CD25- effector T cells (Teff) were isolated from the spleens of DO11.10 mice by using the MACS Treg-cell negative isolation kit (Miltenyi Biotech) as per manufacturer’s instructions. T cells (5 × 10^4^) and irradiated APCs (1 × 10^5^) also isolated from DO11.10 mice were co-incubated with 200 μg/mL OVA and pulsed with 1 μCi of ^3^H at 72 hr. ^3^H incorporation was quantified 16 hrs later by using an automated multi-sample harvester and dry scintillation counter (Packard Instrument Co., Meridan, CT, USA). Data compared against un-stimulated cells were expressed as stimulation index (S.I.). When indicated, the stimulation was performed in the presence of anti-DLL4 or anti-Jagged1 Ab (20 μg/mL, BioXCell).

### Quantitative real-time PCR

For gene expression, cDNA was synthesized by RT-PCR and gene expression was amplified by sequence-matched primer pairs (Table 1) and SYBR Supermix (Roche, NJ, USA) in an Applied Biosystems 7900 real-time PCR system (San Francisco, CA, USA). The data was normalized with the expression of GAPDH or cyclophilin.

### Flow cytometry

Cells were incubated with fluorescence-conjugated Ab or isotype-matched control Ab for 30 min at 4 °C. For intracellualr cytokine staining, cells finishing surface staining were fixed with 4% paraformaldehyde and permeabilized by 0.1% saponin in PBS before cytokine staining. The samples were analyzed with a flow cytometer and CellQuest software (FACScalibur, BD, San Jose, CA). Fluorescence-conjugated antibodies were purchased from eBioscience and BioLegend (San Diego, CA, USA.)

### Western blotting

Cytoplasmic and nuclear proteins were extracted by using the NE-PER extraction reagents as per manufacturer’s protocol (Thermo Scientific). The extracted proteins were separated on 8% gels by SDS-PAGE, transferred onto PVDF membranes (EMD Millipore, MA, USA), and followed by sequential incubation with an primary Ab against Notch intracellular domain (NICD, Cell Signaling, MA, USA) and an HRP-conjugated secondary Ab. Lamin B and GAPDH (Upstate, New York, USA) were used as internal controls for nuclear and cytoplasmic proteins, respectively. Blots were detected by using ECL Plus (Amersham Pharmacia, NJ, USA). Integrated band density was measured by Alpha Innotech Chemilmager 5500 (Alpha Innotech, CA, USA).

### Statistical analysis

Data were expressed as Mean ± SD. Statistical comparisons between groups were made by one-way ANOVA, or unpaired t-test by using GraphPad Prism 5 Software. A *P* value of less than 0.05 was considered as statistically significant.

## Additional Information

**How to cite this article:** Huang, M.-T. *et al*. Notch Ligand DLL4 Alleviates Allergic Airway Inflammation *via* Induction of a Homeostatic Regulatory Pathway. *Sci. Rep.* **7**, 43535; doi: 10.1038/srep43535 (2017).

**Publisher's note:** Springer Nature remains neutral with regard to jurisdictional claims in published maps and institutional affiliations.

## Supplementary Material

Supplementary Information

## Figures and Tables

**Table 1:** Primer sequences.

Gene	Forward primer	Reverse primer
Notch1	CCGTGGCTCCATTGTCTACCT	CATCGGTGGCACTCTGGAA
Notch2	CCAAGCGGAAGCAAGCAT	GGCGCTTGTGATTGCTAGAGT
Notch3	TACTGCATTTGTCCACCTGGAT	TCCAACCTCACCCCCATCT
Notch4	GGTTTGCCAGCTCCTATTGG	CAGCCAGCATCAAAGGTGTAGT
Jagged1	ACCACCTGCGAAGTGATTGAC	GAGATATACCGCACCCCTTCAG
Jagged2	CGTCGTCATTCCCTTTCAGTTC	TCCTCATCTGGAGTGGTGTCAT
DLL1	GGCCAGGTACCTTCTCTCTGATC	GGCGGCTGATGAGTCTTTCT
DLL3	TCCAAGGCTCTAACTGTGAGAAGA	GAGGCCGCCATTCTGACA
DLL4	GACCTGCGGCCAGAGACTT	GCCAAATCTTACCCACAGCAA
T-bet	ACCAGAACGCAGAGATCACTCA	CAAAGTTCTCCCGGAATCCTT
GATA-3	CTACCGGGTTCGGATGTAAGTC	GTTCACACACTCCCTGCCTTCT
ROR-γt	CCGCTGAGAGGGCTTCAC	TGCAGGAGTAGGCCACATTACA
FoxP3	TACCACAATATGCGACCC-3	CTCAAATTCATCTACGGTCC
GAPDH	CTTCACCACCATGGAGAAGGC	GGCATGGACTGTGGTCATGAG
Cyclophilin	ATGTGCCAGGGTGGTGACTTT	TTGCCATCCAGCCATTCAGTC
