# Supplementary material for: Notch Ligand DLL4 Alleviates Allergic Airway Inflammation via Induction of a Homeostatic Regulatory Pathway
Source: Sci Rep. 2017 Mar 6;7:43535. doi: 10.1038/srep43535 (PMC5337933; doi:10.1038/srep43535)

Supplementary Information

Notch Ligand DLL4 Alleviates Allergic Airway Inflammation *via* Induction of a Homeostatic Regulatory Pathway

Miao-Tzu Huang, Yi-Lien Chen, Chia-I Lien, Wei-Liang Liu, Li-Chung Hsu,

Hideo Yagita and Bor-Luen Chiang

Suppl 1. WB used for Fig. 2B

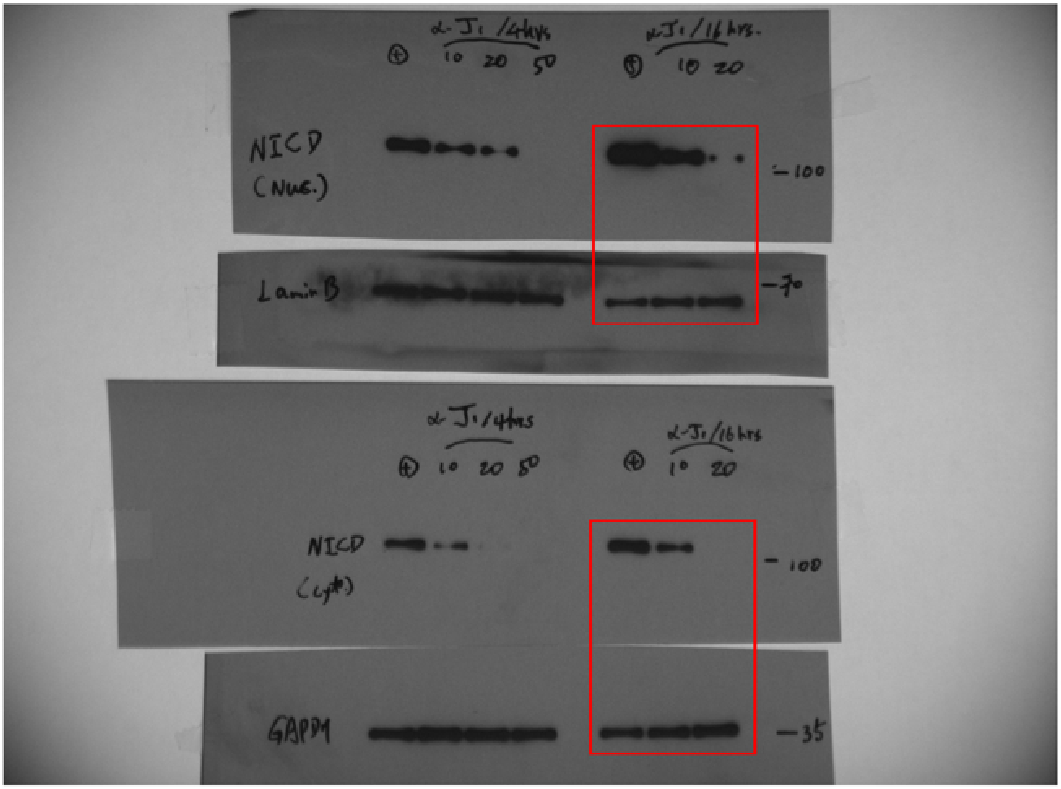

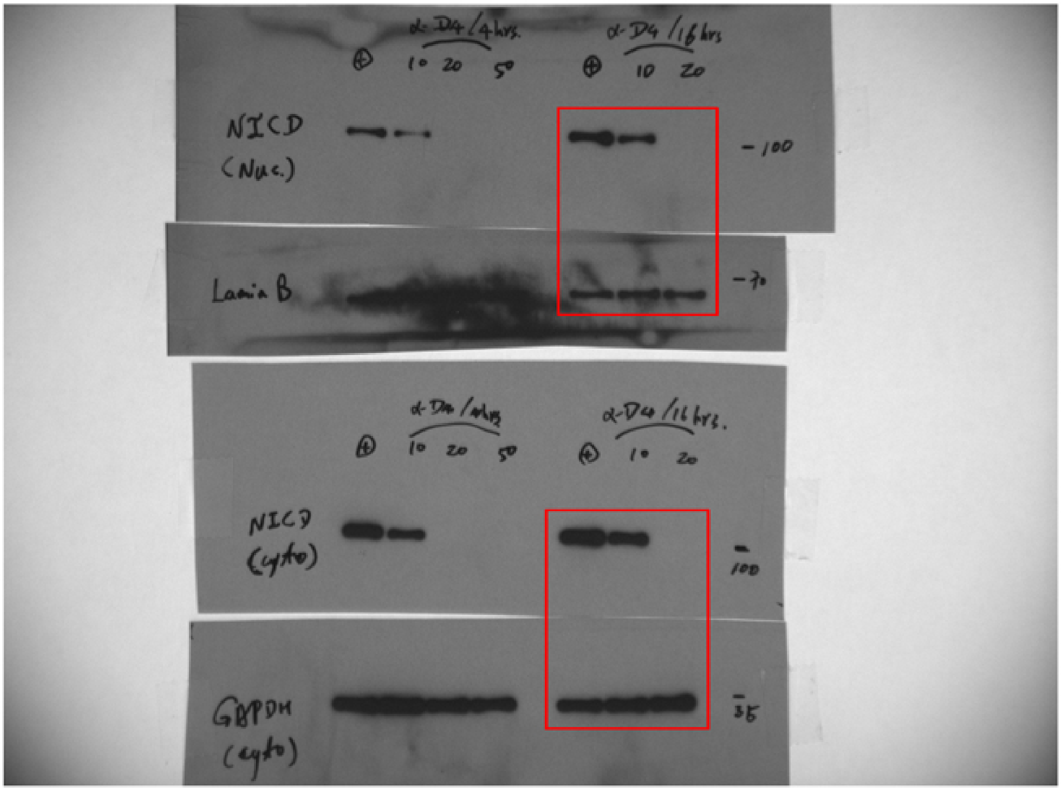

Suppl 2. WB used for Fig. 4A

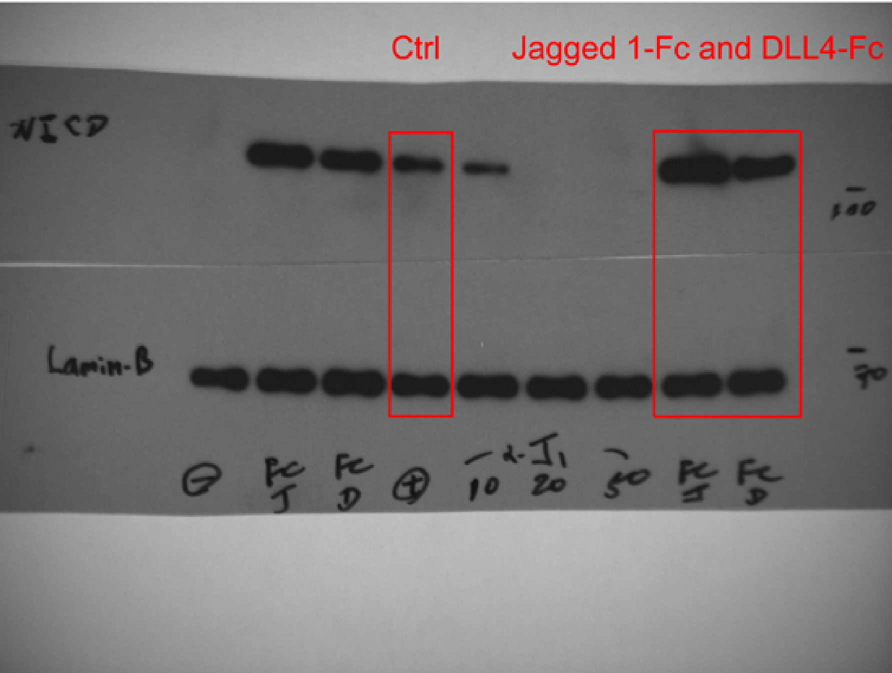

Supplement: Supplementary Information [file srep43535-s1.pdf]
